# Fluorescence Anisotropy as a Temperature-Sensing Molecular Probe Using Fluorescein

**DOI:** 10.3390/mi12091109

**Published:** 2021-09-15

**Authors:** Puneet Jain, Takuya Aida, Masahiro Motosuke

**Affiliations:** 1 Department of Mechanical Engineering, Faculty of Engineering, Tokyo University of Science, 6-3-1, Niijuku, Katsushika-ku, Tokyo 125-8585, Japan; takuya0aida@hotmail.com (T.A.); mot@rs.tus.ac.jp (M.M.); 2 Water Frontier Research Center, Research Institute for Science and Technology, Tokyo University of Science, 1-3, Kagurazaka, Shinjuku-ku, Tokyo 125-8585, Japan

**Keywords:** fluorescence anisotropy, fluorescein, microfluidics, fluorescence polarization, temperature imaging, thermal molecular probe

## Abstract

Fluorescence anisotropy, a technique to study the folding state of proteins or affinity of ligands, is used in this present work as a temperature sensor, to measure the microfluidic temperature field, by adding fluorophore in the liquid. Fluorescein was used as a temperature-sensing probe, while glycerol–aq. ammonia solution was used as a working fluid. Fluorescence anisotropy of fluorescein was measured by varying various parameters. Apart from this, a comparison of fluorescence anisotropy and fluorescence intensity is also performed to demonstrate the validity of anisotropy to be applied in a microfluidic field with non-uniform liquid thickness. Viscosity dependence and temperature dependence on the anisotropy are also clarified; the results indicate an appropriate selection of relation between molecule size and viscosity is important to obtain a large temperature coefficient in anisotropy. Furthermore, a practical calibration procedure of the apparatus constant is proposed. In addition, the potential of temperature imaging is confirmed by the measurement of temperature distribution under focused laser heating.

## 1. Introduction

With the advancement of micro- and nano-technology, microfluidic devices have gained a lot of importance in medical, engineering, and biological fields because of the following advantages: these devices require a small sample volume, the reaction rate is relatively fast, and they are also portable [1,2]. It is well known that thermal transport at micro/nano-scale is different from that at macroscale due to increased surface-to-volume ratio. The thermal behavior in the microfluidic device is governed by heat conduction rather than convection. In addition, the low heat capacity of the system results in a rapid thermal response. Indeed, temperature control in a microfluidic platform is often required to manage many physical, chemical, and biological applications [3]. Furthermore, temperature information in microscopic space around novel nanomaterials is key to evaluate the performance of the photothermal effect in nanomaterials for hyperthermia [4,5]. Additionally, thin liquid film is often used to enhance thermal transport between two solid plates as a thermal interface material (TIM). TIM has a non-uniform thickness in the order of micrometers, corresponding to surface roughness of the plates, resulting in the non-uniform thermal resistance. Temperature imaging at the interface would provide useful insight into the advanced thermal management [6]. Therefore, it is necessary to measure the microfluidic temperature field of a fluid.

To measure the temperature of fluid in a microchannel, microfabricated thermocouples or resistance temperature detectors (RTD) are often used [7]. The problem with them is their relatively large sensor size compared with the channel size. Furthermore, thermocouples or RTD allow us to measure the temperature only at the point of contact. An alternative to micro-thermocouples or RTD is an optical thermal probe, e.g., laser-induced fluorescence (LIF) using fluorescent molecules and thermochromic liquid crystals (TLCs) [8,9,10]. TLCs show different colors depending on the liquid temperature. However, the size of TLC particles needs to be at least 5 μm [11], which is relatively large when considering the microfluidic application. LIF is better than micro-thermocouples/RTD as it is a contactless technique that relies on thermal quenching of fluorescent emission from probe molecules [8,9,10,11,12,13]. LIF also has drawbacks, such as the LIF signal depends on the distribution of numbers of fluorescent probe molecules in the liquid. If the concentration of the probe molecule is non-uniform, then the fluorescence emission is also non-uniform. An alternative to LIF is two-color LIF [14]. This method utilizes temperature-sensitive and temperature-insensitive fluorophores, then the intensity from the temperature-insensitive probe is used to compensate for the non-uniformity of illumination light and liquid thickness. However, two-color LIF uses an assumption that there is uniform concentration of two fluorescent dyes in the microfluidic system. In this sense, multiple fluorophore-based thermometry has a disadvantage.

In this present work, we focus on fluorescence anisotropy as an indicator to measure the temperature of a liquid in a microchannel. Other research groups have used fluorescence anisotropy or polarization to evaluate the folding state of proteins [15,16,17]. Fluorescent anisotropy has advantages over LIF, as fluorescence anisotropy is not affected when there is non-uniformity in molecule concentration, incident light intensity, or liquid thickness, because in fluorescence anisotropy, the signal is normalized by the total output light intensity [18]. Based on this nature of normalization, the anisotropy is suitable to be applied as a thermal indicator in a non-uniform field of illumination, concentration, and liquid thickness. The present work investigates the potential of the fluorescence anisotropy of fluorescein as a molecular thermal probe in a microfluidic platform.

**Figure 1:**
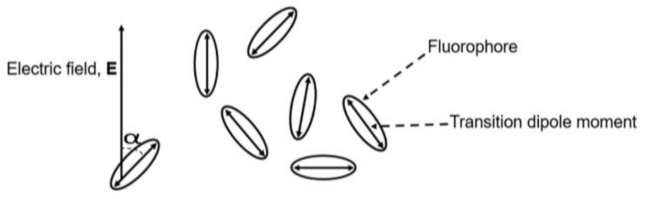
Schematic representation of photo-selection.

**Figure 2:**
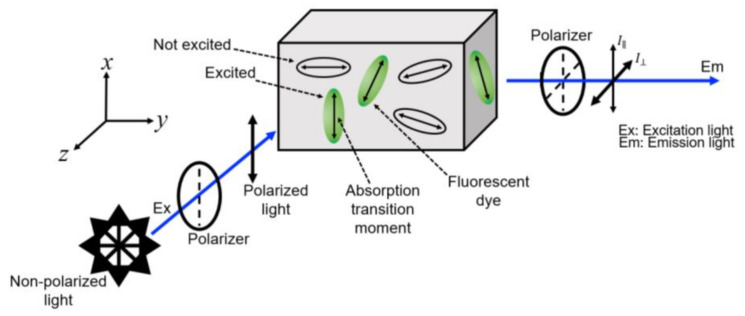
Schematic of fluorescence anisotropy.

Fluorescence Anisotropy: Unpolarized light, after passing through a polarizer, becomes linearly polarized. This linearly polarized light is allowed to pass through a sample containing fluorophores, i.e., fluorescent molecules. Fluorescent molecules have transition dipoles associated with them. The molecules whose transition moments are parallel with respect to incoming polarized light become excited to the higher state. The probability that a molecule becomes excited is proportional to cos^2^*α* (where *α* is the angle between the electric field vector of the incident light and the transition dipole moment of the fluorophore, Figure 1), and this process is known as “photo-selection”. When the excited molecule comes back to the ground state, it emits fluorescence. At the output we measured the parallel (*I*_∥_) and perpendicular (*I*_⊥_) components of the light, where parallel and perpendicular means parallel and perpendicular with respect to the incoming light. If the incoming light is polarized in *x*-direction, then at the output *I*_∥_ = *I_x_* and *I*_⊥_ = *I_y_* = *I_z_*. This is shown in Figure 2. Mathematically, fluorescence anisotropy (*r*) is given using [19,20,21]:

(1) $$r=\frac{I_{∥}-I_{⊥}}{I_{x}+I_{y}+I_{z}}=\frac{I_{∥}-I_{⊥}}{I_{∥}+2I_{⊥}},$$

So, we can see that *r* is normalized by the total output intensity (*I_x_* + *I_y_* + *I_z_*).

Apart from Equation (1), we also define Perrins’s equation and Stokes–Einstein equation as in [22]:

Perrin’s equation:

(2) $$\frac{r_{0}}{r}=1+\frac{\tau }{\theta },$$

Stokes–Einstein equation:

(3) $$\theta =\frac{\eta V}{k_{B}T},$$

where *r*_0_ is the limiting anisotropy (anisotropy without any molecular rotation, also known as freezing anisotropy; discussed in detail, later), *τ* is fluorescent lifetime, *k*_B_ is Boltzmann constant, *T* is temperature of the solution, *V* is molecular volume, and *η* is solution viscosity, respectively. Merging Equations (2) and (3), Perrin’s equation can be converted as a function of the fluid temperature.

(4) $$r=\frac{r_{0}}{1+\frac{\tau k_{B}T}{V\eta }},$$

Equation (4) shows that there is a direct relation between the inverse of anisotropy and temperature. So, if we can know the anisotropy, then we can easily evaluate the temperature and vice versa.

## 2. Materials and Methods

To measure fluorescence anisotropy and fluorescence intensity, fluorescein was used as a thermal probe in the present work. Fluorescein was selected due to the fact that it is a very mature fluorescent dye and researchers have been using fluorescein, along with its derivatives, for years [23,24,25,26,27,28,29]. In addition, it is important to note that fluorescein does not diffuse into polydimethylsiloxane (PDMS); the most common material used for the fabrication of a microchannel.

As stated above, an excited molecule, while coming back to the ground state, experiences either molecular rotation or transfers energy to the other molecules (energy transfer to the other molecules is known as Förster resonance energy transfer (FRET)). Molecular rotation and FRET are major sources of depolarization [20,22,30]. To avoid these, one way is to increase the viscosity of the solution; to achieve this glycerol was used in the present work, and fluorescein solution was prepared by dissolving fluorescein in glycerol–aq. ammonia solution. Aq. ammonia solution (or ammonium hydroxide) was used because of its basicity (pH). Fluorescein shows high fluorescence in basic solutions [31]. A typical concentration of fluorescein is around 0.01 to 1 mM, in this work. 

Fluorescein, glycerol, and aq. ammonia (or ammonia water) were purchased from Wako. Fluorescence anisotropy was measured by two instruments, one was a spectrofluorometer (JASCO FP8300) and another was an optical fluorescence microscope (NIKON Eclipse TE-2000 U); both were customized with rotational polarizers both in the excitation and emission optical paths. The objective lens in the microscope has magnification of 20× and a numerical aperture of 0.45. For anisotropy measurements using a spectrofluorometer, a typical cuvette with an optical path of 10 mm was used and no special arrangement was made. for anisotropy measurements using an optical fluorescence microscope, the working fluid was -seed in a microchannel fabricated using polydimethylsiloxane (PDMS) by soft lithography. The microchannel has a non-uniform depth, equivalent to the optical path, around 20 to 40 μm. To study the temperature sensitivity, fluorescein isothiocyanate–dextran (FITC–dextran) was also used, apart from fluorescein. FITC–dextran was purchased from Sigma. This is because the volume of FITC–dextran is 100 times that of fluorescein. In the measurement of temperature field under local heating, a diode laser (COHERENT OBIS 640 LX) was used as a heating source. To enhance the light absorption of the laser beam, brilliant blue FCF, purchased from Wako, was added to the aqueous solution. The excitation wavelength for a spectrofluorometer and an optical microscope is 480 nm and 450–490 nm, and the emission spectral band for a spectrofluorometer is 400–700 nm, while that for the optical microscope is 500–550 nm. 

## 3. Results and Discussion

**Figure 3:**
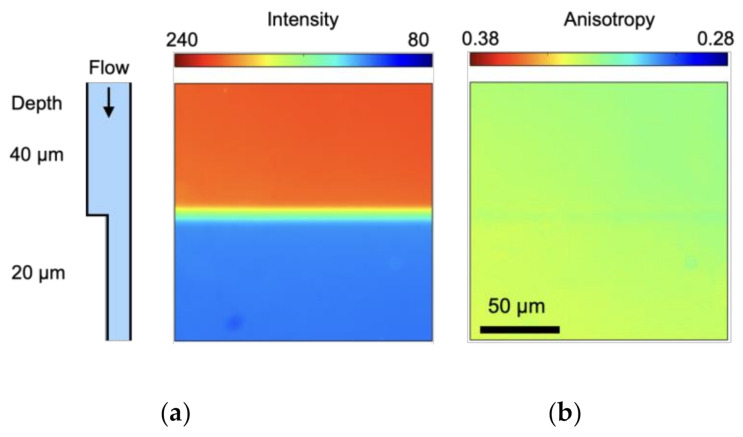
(**a**) Fluorescence intensity and (**b**) fluorescence anisotropy for fluorescein solution in a microchannel with different depths.

As stated in the introduction, fluorescence anisotropy is independent of a sample’s depth. To confirm this, fluorescence intensity and anisotropy of fluorescein solution in a depth-varying microchannel were measured, where the depth of the microchannel varied from 40 to 20 μm. The result is shown in Figure 3. From Figure 3a, it is seen that with varying the depth, fluorescence intensity varies. This is due to the decrease of optical path length. Since the concentration of the dye is constant in 40 and 20 μm depth, but the channel height decrease, which decreases the fluorescence intensity. As number of particles in smaller area are more so this leads to quenching.

Furthermore, it can be clearly seen that, irrespective of a sample’s depth, anisotropy is nearly uniform with a standard deviation of 0.0037, while intensity almost doubles with channel depth. This is because fluorescence anisotropy is normalized by the total intensity (Equation (1)). Tatsumi et al. utilized fluorescence polarization *P* (Equation (5)) to measure the temperature of a liquid in a microchannel [32].

(5) $$P=\frac{I_{∥}-I_{⊥}}{I_{∥}+I_{⊥}},$$

However, polarization and anisotropy are different in the sense that anisotropy is normalized by the total intensity, whereas polarization is not normalized. Polarization can become a useful parameter of thermal indicators only in the spatially uniform case in terms of molecular concentration, light intensity, and channel height. Therefore, it can be stated that the fluorescent anisotropy is a more versatile parameter in the use of non-uniform situations; rough surface, light illumination intensity, and dye concentration.

**Figure 4:**
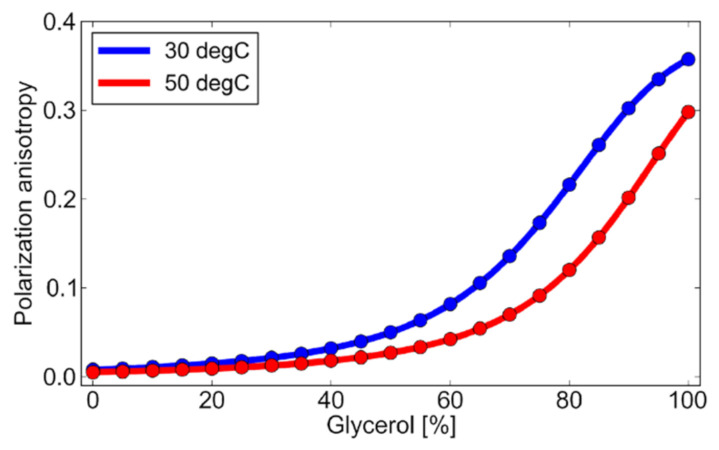
Fluorescence anisotropy of fluorescein solution as a function of a solution’s viscosity.

Figure 4 shows the anisotropy of fluorescein solution by varying the glycerol concentration, measured at 30 and 50 °C. Figure 4 shows that as the glycerol percentage is increased, i.e., as the solution becomes more viscous, the anisotropy increases. This is because as the viscosity of the solution increases, the molecular rotation of the excited molecules, coming back to ground state, is reduced by the viscous nature of the solution. Molecular rotation is a source of depolarization [22], as also discussed above. As in Equation (4), the anisotropy is sensitive to the liquid temperature, the viscosity, and the lifetime. In an isothermal system, the anisotropy is utilized as a viscous indicator [33,34]. Here, we intend to utilize the temperature dependence of the anisotropy to measure the liquid temperature field in microfluidics. 

**Figure 5:**
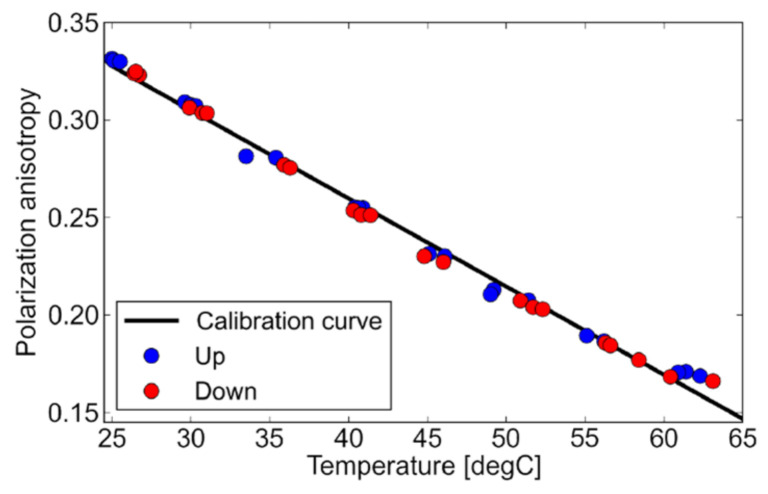
Fluorescence anisotropy of fluorescein solution as a function of temperature.

The temperature dependence of fluorescence anisotropy of 80% glycerol solution in a wider temperature range from 25 to 65 °C is shown in Figure 5, where a calibration curve up and down means the data obtained in the heating and cooling process, respectively. As the temperature increases, fluorescence anisotropy almost linearly decreases. This is because with an increase in solution temperature, molecular rotation increases, and as stated above, molecular rotation is a source of fluorescence depolarization. Figure 5 shows that fluorescence anisotropy showed a negative temperature dependence, and the temperature coefficient is −4.5 mK^−1^. A slight difference from the linear correlation would be the influence of viscosity reduction due to the temperature increase. However, this influence is not significant considering the application of fluorescein as the temperature measurement in this range.

**Figure 6:**
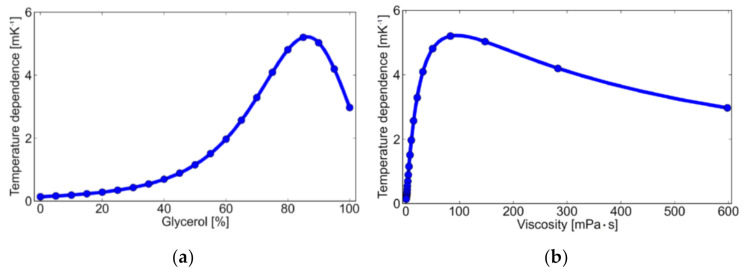
Temperature dependence (absolute value of temperature coefficient) of fluorescein as a function of (**a**) glycerol percentage and (**b**) viscosity of the solution.

Figure 6 shows the temperature dependence of fluorescein as a function of glycerol percentage and solution viscosity. Figure 6 is just a derivative of data shown in Figure 4. Note that the temperature coefficient itself is negative and the dependence shown in the Figure is an absolute value. From the results, the temperature dependence indicated a peak around a glycerol percentage of 85%, i.e., viscosity of 80 mPa·s. It shows that we need to pay attention to the relationship of probe size and solution viscosity. In water (~1 mPa·s), the temperature coefficient is −1.4 mK^−1^. When we apply fluorescein as a thermal probe in water-based microfluidics, the suppression of rotational motion of the molecule by the use of large molecules works. In this study, we investigated fluorescein isothiocyanate–dextran, whose volume is about 100 times greater than fluorescein. The measurement results show that the fluorescein-labeled dextran has a temperature coefficient of −8.9 mK^−1^, 6.3 times larger than that of normal fluorescein. Therefore, we can adjust temperature sensitivity by fluorescent labeling depending on the viscosity of the liquid.

**Figure 7:**
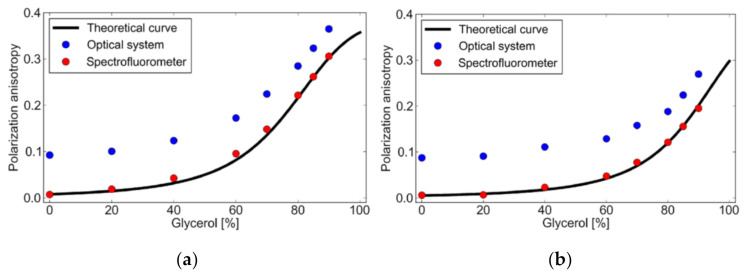
Theoretical and measured fluorescence anisotropy values at (**a**) 30 °C and (**b**) 50 °C.

**Figure 8:**
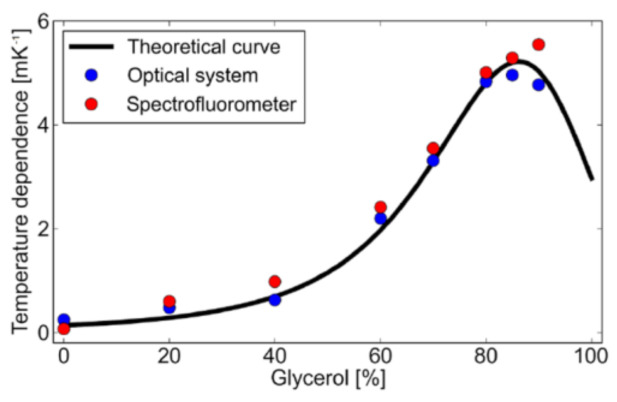
Temperature dependence of theoretical and measurement anisotropies (absolute values).

Anisotropy measured using a spectrofluorometer and fluorescence microscope was compared with that of anisotropy values obtained theoretically using the converted Perrin’s equation (Equation (4)). To calculate the anisotropy, the physical parameters shown in Table 1 were used, while the used temperatures were 30 °C (Figure 7a) and 50 °C (Figure 7b). Viscosity was calculated using the method provided by Cheng et al. [35]. It is seen that the theoretical values at both temperatures match well with the measurement results from the spectrofluorometer. There is a great discrepancy between the values obtained by the spectrofluorometer and optical microscope. The microscope shows larger anisotropy than the spectrofluorometer from 0.07 to 0. Both 30 and 50 °C measurements show the same trend. This could be because of homo-FRET, due to different dimensions of the sample holder. The more the excited-state molecule interacts with the homo-molecules, the more FRET will happen. For the spectrofluorometer, the sample holder is a 10 mm-thick cuvette (three-dimensional), while for the optical microscope, it is a microchannel (two-dimensional). In the cuvette, the excited-state dye has a higher possibility of interacting with more molecules for the energy transfer, but in a microchannel the possibility is reduced. So, the higher the FRET, the lower the anisotropy, as FRET is a source of depolarization. Figure 8 shows the temperature dependence of fluorescence anisotropy for theoretical and measured values. It is clearly shown that the effect of the optical system on the fluorescent anisotropy is canceled when we just consider its temperature dependence. This fact helps to establish a simple calibration procedure in the temperature measurement. One may measure the fluorescent anisotropy at a temperature, e.g., room temperature, to obtain the reference value, which depends on the optical system, and then use it for the theoretical estimate of temperature.

So, from the above experiments it can be said that fluorescence anisotropy of fluorescein can be a potential tool to measure the microfluidic temperature, just by adding fluorophore (fluorescein with appropriate labeling) to the liquid. Of course, this method can eliminate the need for sensors, or possess other drawbacks that are discussed in the Introduction section. The other advantage of fluorescent anisotropy as the liquid temperature measurement is cost effectiveness. One can just add two rotational polarizers in a fluorescent microscope.

**Figure 9:**
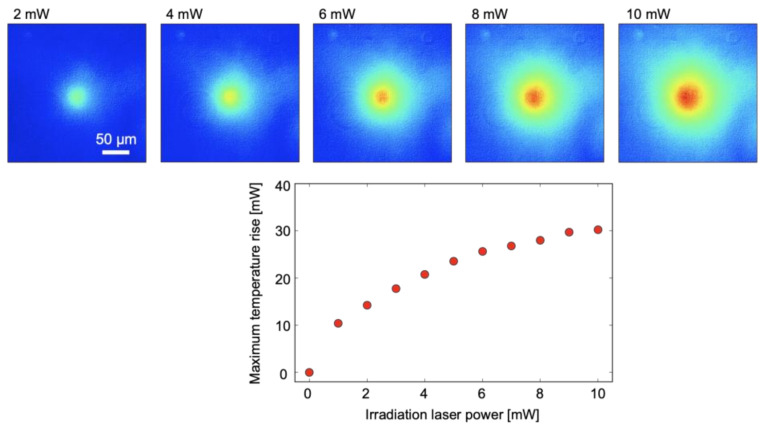
Temperature measurement results under local laser heating. (**upper**) Temperature fields at 2, 4, 6, 8, and 10 mW, (**lower**) maximum temperature rise at various laser powers.

As a proof of concept, temperature measurement of water under local heating by a focused laser beam was performed. The laser spot size in the microchannel was approximately 10 μm. Here, FITC–dextran with a concentration of 0.1 mM was added to water, and brilliant blue FCF was also added to the solution. Figure 9 depicts the measurement results. It is apparent that the temperature field has a peak around the center of the image, and that the temperature rise corresponds to the optical irradiation power. The measured temperature distributions are concentric; this is typical thermal behavior in the low-thermal Peclet number system (almost equivalent to low Reynolds number flow). The conduction-dominated temperature distribution shows the validity of the measurement.

## 4. Conclusions

Fluorescence anisotropy of fluorescein has potential to be a good candidate to measure the temperature of a fluid in a microscopic domain. As the temperature of the fluid increases from 25 to 65 °C, the anisotropy of fluorescein decreases, due to the increased molecular rotation with an increase in temperature. It has been found that fluorescence anisotropy of fluorescein shows a negative temperature dependence with a temperature coefficient of −4.5 mK^−1^ in 80% glycerol solution, while the coefficient can be tunable with appropriate labeling which matches the solution viscosity. The fluorescein-labeled dextran (FITC–dextran) shows a temperature dependence of −8.9 mK^−1^ in water; this temperature coefficient is 6.3 times larger than normal fluorescein. As the viscosity of the solution increases, molecular rotation of the excited-state molecules reduces, and hence anisotropy increases with a peak. Although the fluorescence anisotropy is affected by an optical system, especially magnification optics, this effect is negligibly small in the temperature sensitivity. The apparatus constant can be calibrated just by the measurement of the anisotropy value in one solution and temperature. Measurement results of temperature distribution under local laser heating demonstrate the potential of fluorescence anisotropy as a novel microfluidic temperature-imaging technique in various applications.

## Figures and Tables

**Table 1:** Physical properties of fluorescein.

Parameter	Value
Limiting anisotropy, *r*_0_	0.38
Fluorescent lifetime, *τ*	3.7 ns
Volume, *V*	0.41 nm^3^
